# Improving Research Patient Data Repositories From a Health Data Industry Viewpoint

**DOI:** 10.2196/32845

**Published:** 2022-05-11

**Authors:** Chunlei Tang, Jing Ma, Li Zhou, Joseph Plasek, Yuqing He, Yun Xiong, Yangyong Zhu, Yajun Huang, David Bates

**Affiliations:** 1 Brigham and Women’s Hospital Harvard Medical School Boston, MA United States; 2 Sichuan Academy of Medical Sciences and Sichuan Provincial People's Hospital Sichuan China; 3 School of Economics Fudan University Shanghai China; 4 School of Computer Science Fudan University Shanghai China

**Keywords:** data science, big data, data mining, data warehousing, information storage and retrieval

## Abstract

Organizational, administrative, and educational challenges in establishing and sustaining biomedical data science infrastructures lead to the inefficient use of Research Patient Data Repositories (RPDRs). The challenges, including but not limited to deployment, sustainability, cost optimization, collaboration, governance, security, rapid response, reliability, stability, scalability, and convenience, restrict each other and may not be naturally alleviated through traditional hardware upgrades or protocol enhancements. This article attempts to borrow data science thinking and practices in the business realm, which we call the data industry viewpoint, to improve RPDRs.

## Introduction

Research Patient Data Repositories (RPDRs, eg, Integrating Biology & the Bedside [i2b2]) and their rapid organic evolution are critical to linking disparate and high-dimensional patient data for a wide range of applications in research. One goal for RPDRs’ evolution in clinical and translational science is to subsume biomedical data science infrastructures and infrastructural health data science [1,2], such as rapid pharmacovigilance [3] and the delivery of real-world evidence at the point of care to actualize the learning health care system [4]. The path to achieving this goal may be tortuous since problems may not emerge until fundamental issues are resolved. Biomedical data science aims to use data technology of any kind to advance medical society as a transdisciplinary ecosystem [4,5] by unifying different disciplines beyond their traditional boundaries to address a common problem. The complexity of the data science ecosystem increases the difficulty of improving RPDRs. Improving RPDRs, therefore, requires a wide variety of new functions and capabilities in the administrative, organizational, and educational areas, including data integration, management, education, support, tooling, governance, optimization, and alignment across missions [3,6].

**Figure 1:**
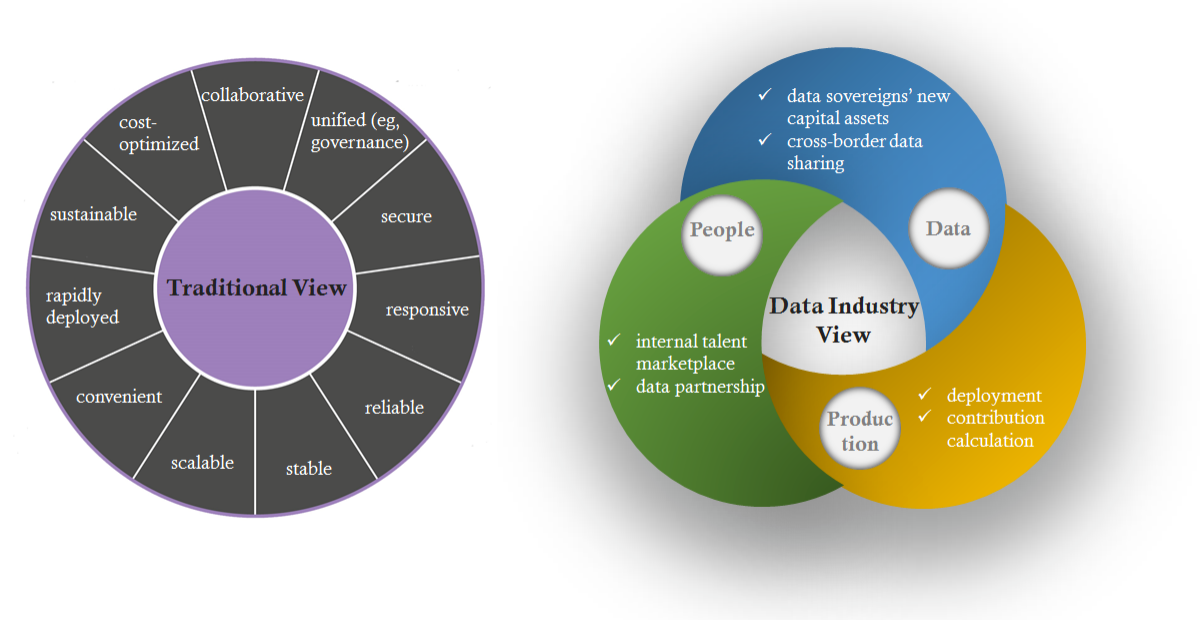
Comparison between traditional and data industry viewpoints of Research Patient Data Repositories.

The effort to establish and sustain biomedical data science infrastructures would benefit if it borrowed thinking and best practices from the data industry [7]. Data industry thinking includes perspectives on data-driven research, innovation, industrialization, and opportunities. We hypothesize that data industry thinking may reshape prevailing views of how people interact with data value and data production in the context of RPDRs (Figure 1).

## Data Production: Deployment Challenges and Contribution Calculations

Data production involves the generation, storage, and curation of data from data-centric human (social, economic, and scientific) activities. Intuitively speaking, it is the process of combining various analyzable data inputs for consumption. The consumption process starts with incoming raw materials used for the preparation of semifinished (eg, pretrained word embeddings) and finished data products (eg, a service). The raw data and data products are “nonrivalrous” in nature, meaning they can be used by multiple users at once without depletion of the resource. Data products can act as reusable resources [8], assets [9], or capital [10] to accelerate research.

When considering data production in RPDRs, some previously unseen problems may arise, such as deployment. Campion Jr et al [11] reported that deployment challenges are widespread in the existing RPDRs: “a number of tools commonly but not uniformly implemented”; for example, i2b2 enables investigators to obtain deidentified patient counts without SQL programming [12]. Many incorrectly think of deploying a data science or analytical model as the last stage of the process. Starting with the algorithm first, and only at the end of the project thinking about how to insert it into the process, is where many deployments fail [13]. Scientists can readily interact with RPDRs to access the underlying electronic health record (EHR) data. RPDRs should additionally provide a solution for fully and successfully implementing analytical and artificial intelligence models from experimentation to production. The first tools to consider to mitigate deployment challenges are tools for handling structured and unstructured EHR data, such as exploratory analysis and data self-governance tools. Exploratory data analysis is an important data industry best practice step focused on gaining insights from raw data prior to training learning models. Exploratory analysis tools that go beyond basic initial data analysis tasks (like SQL programming, ie, sort, filter, aggregate, correlate, group, derive attributes) are essential for handling tasks that previously were manual, heuristic-based, or simply impossible [14]. The transformation of unstructured clinical notes which contain summaries (eg, history of present illness) that describe and illustrate the longitudinal course of specific clinical events or situations experienced by patients into an appropriate data representation (eg, annotated corpus of pretrained word embeddings or a hierarchical representation with multiple levels of granularity) can offer RPDRs enhanced machine intelligence for downstream analysis and reduce duplicated preprocessing efforts to make this data computable [15]. Data self-governance models like Databox [16] can support data sharing that meets study eligibility criteria documented in RPDRs. These default tools can be customized as digital “errand runners” [17] to replace deeply occupational tasks that are tedious, time-consuming, and not artistic.

Data product sharing should be encouraged by the data sovereigns of RPDRs [18], including cross-border data flows. Multilevel data products, such as models, code, intermediate results, annotated training corpora, enclaves, experimental findings, presentations, preprints, and retrieved literature citations can be found throughout the entire life cycle of medical research and are helpful for accelerating complementary efforts. We recommend transplanting contribution margin–based pricing from the data industry to RPDRs to facilitate data sharing. These contributions include but are not limited to reuse frequency, shareable integrity, quantity versus speed in question and answer responses, and compliance practices. Contribution calculations can support employee engagement in the RPDR community and serve as an accelerator for scientific discovery.

## People: Internal Talent Marketplace and Data Partnerships

We suggest that RPDR processes and structures be optimized based on the organizational structure, how stakeholder power is exercised, how stakeholders communicate their needs, how decisions are made, and how decision-makers are held accountable. Data production relies on the efforts of a community of interdisciplinary users, including data scientists, enterprise information technology personnel, clinicians, researchers, informaticists, data engineers, data analysts, annotators, and other data product enhancers. The data partnerships' teams rely on an organization's brand to undertake and complete data production. These teams can freely use RPDR data within organizations, and products or services carried out by these teams will be shared within the company. When the velocity of data partnerships in a market exceeds that of an organization, inefficiencies will cause the organization to lose competitive advantages. As markets evolve, an organization will inevitably choose to focus on cost (ie, replacing human labor with machines) or evolve their organizational structure. Flattening the organizational hierarchy so that people can work together “more equally” will lead to increased efficiencies from equitable data partnerships and the rise of the internal talent marketplace. As an upgraded version of a “principal investigator,” a data partnership might not just rely on grants but also on contributions. In essence, the organization has evolved into a market with relatively small competition. Crowdsourcing within an organization is an alternative for these teams to achieve their goals and with it, the rise of the internal talent marketplace is achieved. The internal talent marketplace takes advantage of the increased flexibility of the gig economy and marketplace-based platforms without requiring changes to employment categories. It matches internal employees and, in some cases, a pool of contingent workers to short-term projects and work. Thus, under ideal next-generation RPDRs, these trends among employees can result in collaborative translational medicine by maintaining an innovation ecosystem through teamwork, trust, reliability, and collaboration.

## Conclusions

Best practices in RPDRs tend to focus on core infrastructural and methodological needs, such as machine-readable standards, data access platforms, search and discoverability, claim validation, and insight generation [19]; we argue that the complementary data industry viewpoint is relevant and apposite. From this point of view, RPDRs must consider production deployment and contribution calculations, the establishment of internal talent marketplaces and data partnerships, as well as data sovereigns’ new capital assets and cross-border data sharing, as they reveal issues that are not typically addressed. Only with innovative deployed tools, the wide availability and use of diverse data products, and achievable foresight will the future of ideal next-generation RPDRs be truly accessible.

## Abbreviations

EHR: electronic health record
RPDR: Research Patient Data Repositories
